# Fine Root Density Dynamics and Carbon Stock of *Eucalyptus* spp.: Interplay of Age, Genotype, and Edaphoclimatic Conditions

**DOI:** 10.3390/plants13111503

**Published:** 2024-05-30

**Authors:** Josiana Jussara Nazaré Basílio, Otávio Camargo Campoe, Túlio Barroso Queiroz, Cléber Rodrigo de Souza, Rafaela Lorenzato Carneiro, Clayton Alcarde Alvares, Marco Aurélio Figura

**Affiliations:** 1 Department of Forestry Sciences, Federal University of Lavras (UFLA), Lavras 37200, Brazil; 2 Department of Forestry Sciences, São Paulo State University “Júlio Mesquita Filho” (UNESP), Botucatu 18600, Brazil; 3 Institute of Forestry Research and Studies (IPEF), Piracicaba 13415, Brazil; 4 Warnell School of Forestry & Natural Resources, University of Georgia, Athens, GA 30602, USA; 5 Suzano SA, Limeira 13473, Brazil; 6 Klabin, Telêmaco Borba 84261, Brazil

**Keywords:** clone, soil water availability, fine root biomass, deep soil, carbon stock

## Abstract

Roots play a fundamental role in forest ecosystems, but obtaining samples from deep layers remains a challenging process due to the methodological and financial efforts required. In our quest to understand the dynamics of *Eucalyptus* roots, we raise three fundamental questions. First, we inquire about the average extent of the roots of two contrasting *Eucalyptus* genotypes. Next, we explore the factors that directly influence the growth and depth of these roots, addressing elements such as soil type, climate, and water availability. Lastly, we investigate how the variation in *Eucalyptus* species may impact root growth patterns, biomass, and carbon stock. In this study, we observed that the maximum root depth increased by an average of 20% when genotypes were grown on sites with higher water availability (wet site). *E. urophylla* stands had a higher biomass and carbon stock (5.7 Mg C ha^−1^) of fine roots when cultivated on dry sites (annual rainfall~727 mm) than the wet sites (annual rainfall~1590 mm). In *E. grandis* × *E. camaldulensis* stands, no significant differences were observed in the stock of fine root biomass (3.2 Mg C ha^−1^) between the studied environments. Our results demonstrated that genotypes with greater drought tolerance (*E. grandis* × *E. camaldulensis*) tend to maintain higher stocks of fine root biomass (3.2–6.3 Mg ha^−1^) compared to those classified as plastic (*E. urophylla*), regardless of the edaphoclimatic conditions of the cultivation site. Finally, our research helps understand how Eucalyptus trees adapt to their environment, aiding sustainable forest management and climate change mitigation. We also provide a practical tool to estimate underground biomass, assisting forest managers and policymakers in ensuring long-term forest sustainability.

## 1. Introduction

While wood and stems are commonly utilized for lumber and paper production, roots can be repurposed for biomass energy or biochar, with the potential for subsequent sale to plant production companies [1]. Understanding and accurately estimating root biomass is crucial for optimizing resource utilization and informing sustainable land management practices. A comprehensive understanding of root growth and development patterns in deep soil layers is crucial for understanding ecosystem functioning as these processes heavily rely on the roots [2,3]. However, few studies have been carried out to understand the belowground biomass stock. This trend is understandable given the methodological complexity of exploring deeper soil layers, necessitating a highly skilled workforce to handle equipment and identify and process fine roots in the laboratory [4].

Fine roots, typically defined as those with a diameter equal to or less than 2 mm, play essential roles in tree growth and development, including water and nutrient uptake [3,5]. They constitute the active part of the root system concerning carbon sequestration through processes such as respiration, exudation, decomposition, and carbon synthesis [6]. Although fine roots are more abundant in the upper one meter of soil [7,8,9], with a biomass stock ranging from 0.2 to 5 kg m^−2^ [10], their presence has been observed in deeper layers in tropical and subtropical regions [11]. For instance, ref. [11] found fine roots at a depth of 17 m in 3.5-year-old *Eucalyptus* plantations in Brazil. Recent studies also demonstrate that about 50% of the fine root biomass of trees is located at depths greater than 1 m [9,11].

Fine roots growing at depths greater than 5 m play a crucial role in increasing the amount of available water for trees and coping with seasonal droughts [4,11,12,13]. This is because, compared to surface roots, deep roots have larger diameter vessels and tracheids, which increase hydraulic conductivity [14], as well as the likelihood of absorbing water from the groundwater table [15]. Therefore, characterizing the distribution of deep roots in contrasting ecosystems is essential for improving the description of ecosystem services [2], such as mitigating the effects of climate change through carbon capture and storage in the soil.

In addition to water availability, other factors such as fertility [12], soil texture [14], stand age [11], and intrinsic species characteristics [9] can also influence the architecture, density, and maximum depth of root systems. Although we have some general knowledge of the vertical distribution of fine tree roots, further information on their growth and development is still needed for many species [16]. Typically, species with growth characteristics that allow for greater drought tolerance, such as *E. grandis* × *E. camaldulensis*, tend to allocate more carbon to the formation of belowground organs compared to plastic genotypes like *E. urophylla*, which have high productivity [17,18]. However, studies comparing deep rooting patterns of genotypes with distinct growth characteristics are scarce. Therefore, our focus is on exploring the effects of age and edaphoclimatic conditions on the vertical distribution and biomass stock of the fine roots of commercially planted *Eucalyptus* genotypes in Brazil.

Despite this region being considered the most productive in the world [19], with an average annual increment of 32.7 m ^3^ ha^−1^ year^−1^, in recent years, the rate of productivity increase has decreased by about 6 m ^3^ ha^−1^ year^−1^ [20]. This reduction is due to the increasing expansion of crops into marginal regions characterized by extreme climatic conditions, such as high temperatures and low precipitation levels [21]. Our research provides insights into the various growth strategies adopted by *E. grandis* × *E. camaldulensis* (drought-tolerant) and *E. urophylla* (plastic or generalist) genotypes, as well as information that can guide more sustainable and effective forest management practices in a scenario of increasingly evident climate change and environmental pressures. In summary, this study aims to explore how cultivation site and age impact root depth and fine root biomass in *Eucalyptus* clones, with the expectation of observing increased fine root biomass in regions characterized by lower precipitation levels.

## 2. Results

### 2.1. Compartmentalized Carbon Stock According to Site, Age, and Genotype

The *Eucalyptus* plantations stored an average of 38.7 Mg C ha^−1^ (leaves, wood, coarse roots, and fine roots combined) at three years of age. This value represents, on average, 52% of the total carbon stored in these components at six years of age, i.e., at the end of the rotation (81.2 Mg C ha^−1^). The carbon stock in the leaves, wood, coarse roots, and fine roots at six years of age was 1.5, 61.5, 15.5, and 2.7 Mg C ha^−1^, respectively; that is, 4% of the carbon stock was in the fine roots. Considering that at the end of the rotation, the stems are removed from the stands, our results demonstrate that, on average, 19.7 Mg C ha^−1^ was incorporated into the ecosystem, of which 13% corresponded to the carbon stock in the fine roots (Table 1).

Both genotypes exhibited a significant increase in the total biomass stock from the middle (3 years) to the end of the rotation (6 years). The total biomass stock of *E. urophylla* increased by 51–52% and that of *E. grandis* × *E. camaldulensis* increased by 38–61% at the dry and wet sites, respectively. Additionally, site had a positive effect on the total biomass stock, with higher values measured at the wet site (annual rainfall~1590 mm), reaching 101.9 and 209.2 Mg ha^−1^ for *E. urophylla* and 76.9 and 198.9 Mg ha^−1^ for *E. grandis* × *E. camaldulensis* at three and six years of age, respectively. At the dry site (annual rainfall~727 mm), values of 74.5 and 151.4 Mg ha^−1^ and of 56.0 and 101.8 Mg ha^−1^ were recorded for *E. urophylla* and *E. grandis* × *E. camaldulensis* at three and six years of age, respectively. *E. urophylla* had the highest total biomass stocks regardless of site or age. This suggests a consistent trend for this genotype to accumulate significant amounts of biomass over time, with greater adaptability to the edaphoclimatic conditions of the cultivation site (Table 1).

**Figure 1:**
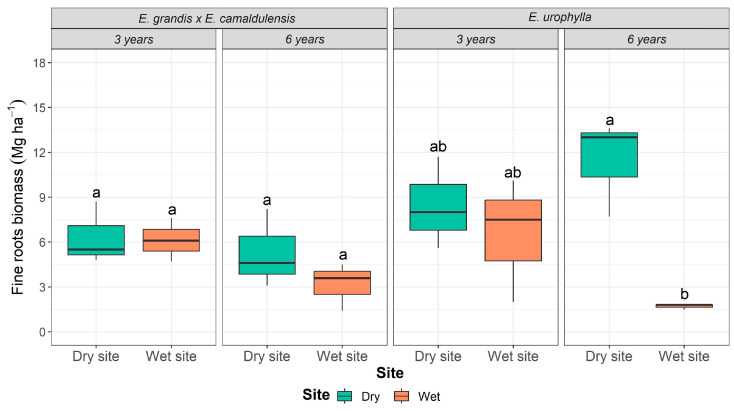
Accumulated fine root biomass (Mg ha^−1^) in the *E. urophylla* and *E. grandis* × *E. camaldulensis* genotypes at three and six years of age when grown on dry and wet sites. Different lowercase letters indicate statistical differences for the combined genotype and site variables at 5% probability.

Age and site influenced the fine root biomass stock of both genotypes. At sites with reduced water availability (annual rainfall~727 mm), there was a 26% increase in the fine root biomass stock of *E. urophylla* from three to six years. However, the fine root biomass stock of this genotype was reduced by 20% at the wet site. By contrast, the fine root biomass stock of *E. grandis* × *E. camaldulensis* showed no significant differences over time (Figure 1).

The fine root biomass stock was generally higher at the dry site regardless of age or genotype. Significant changes were found for *E. urophylla*, which exhibited values between 8.43 and 6.55 Mg ha^−1^ for the fine root biomass stock at three years of age, while at six years of age, the values were 11.42 and 1.67 Mg ha^−1^ at the dry and wet sites, respectively. On the other hand, *E. grandis* × *E. camaldulensis* presented a fine root biomass stock of 6.33 and 6.15 Mg ha^−1^ at three years of age and 5.29 and 3.18 Mg ha^−1^ at six years of age at the dry and wet sites, respectively (Figure 1).

### 2.2. Vertical Distribution of Fine Root Density

**Figure 2:**
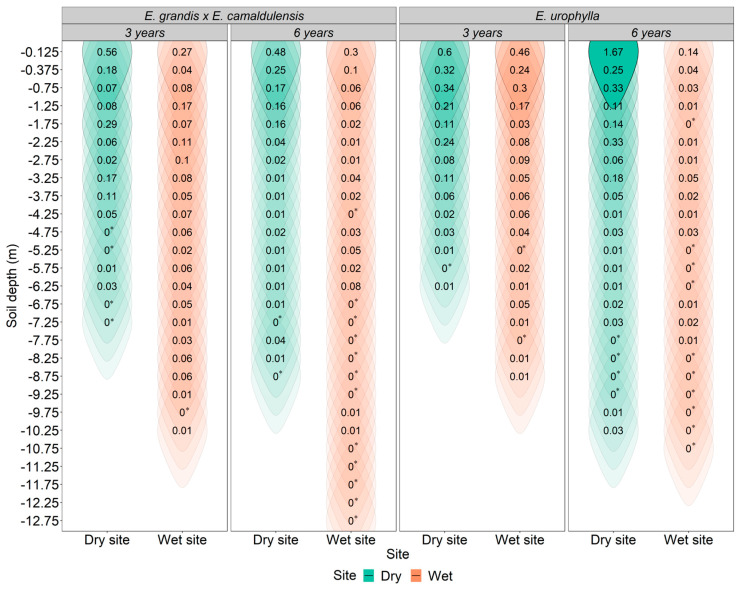
Distribution of fine roots to the maximum depth reached for the *E. urophylla* and *E. grandis* × *E. camaldulensis* genotypes at three (3) and six (6) years of age when cultivated on dry and wet sites. * Fine root density is less than 0.01 g dm^−3^.

The *E. urophylla* and *E. grandis* × *E. camaldulensis* genotypes exhibited high densities of fine roots in the surface soil layers (up to 1 m) regardless of age or cultivation site, with values between 0.03 and 1.67 g dm^−3^. These values represent, on average, 54% of the total fine root density throughout the soil profile (Figure 2).

*E. urophylla* exhibited a higher cumulative density of fine roots in the superficial soil layer (<1 m) compared to *E. grandis* × *E. camaldulensis*, with a 36% and 60% increase at the dry and wet sites, respectively, at 3 years of age, and a 61% increase at the dry site at 6 years of age. However, this trend was not observed at the wet site at 6 years of age as the cumulative density of fine roots in the first meter of soil was 55% higher for *E. grandis* × *E. camaldulensis* than for *E. urophylla*. Approximately 30% of the total fine root biomass occurred in the intermediate soil layer (between 1 and 5 m). Although fine roots were found at greater soil depths, it is worth noting that only about 10% of the total fine root density was found at depths greater than five meters. Furthermore, *E. grandis* × *E. camaldulensis* exhibited the highest fine root density in very deep soil layers (11%) compared to *E. urophylla* (6%). Understanding how different *Eucalyptus* genotypes respond to soil and water conditions can provide valuable information for forest management and the selection of species best suited to specific environmental conditions.

### 2.3. Root Depth and Height Growth

**Figure 3:**
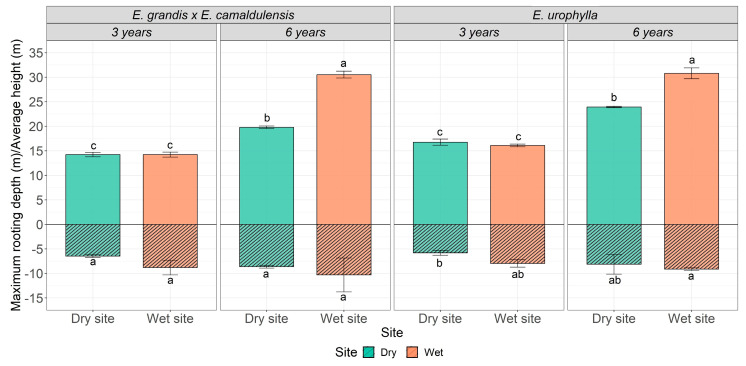
Average height of trees and maximum rooting depth of *E. urophylla* and *E. grandis* × *E. camaldulensis* genotypes at 3 and 6 years of age at the dry and wet site. Upper letters refer to the height variable, and lower letters refer to the root depth.

The root depth and height of the trees were positively influenced by age and cultivation site. Regardless of the genotype, the tallest trees were found at 6 years of age at the wet site, where *E. urophylla* reached a height of 30.8 m and *E. grandis* × *E. camaldulensis* reached 30.5 m. These values are 22% and 35% higher than the values obtained at three years of age for *E. urophylla* and *E. grandis* × *E. camaldulensis*, respectively, at the wet site. Thus, the smallest values were measured at three years of age, i.e., 14.2 m for *E. grandis* × *E. camaldulensis* at both sites and 16.1 m and 16.8 m for *E. urophylla* at the wet and dry sites, respectively. However, when analyzing the genetic materials, we found that, regardless of site or age, *E. urophylla* exhibited the greatest height (Figure 3).

We did not find significant differences in the root depth of *E. grandis* × *E. camaldulensis* based on age and cultivation site. However, *E. urophylla* exhibited a similar behavior to what was found for height, showing an increasing trend in root depth with the age of the stand. The greatest root depth was found for this genotype at the wet site (9.1 m), followed by the dry site (8.1 m) at six years of age. At three years of age, however, the maximum root depth of *E. urophylla* was 29% and 13% lower than the values found at six years of age at the dry and wet sites, respectively (Figure 3). However, when comparing this variable for the different genotypes, we noticed an opposite behavior to what was found for height, where the greatest root depths were found for the *E. grandis* × *E. camaldulensis* genotype, partially corroborating our third hypothesis (Figure 3).

The vertical extensions of the trees (height × root depth) did not show symmetry below and above the ground regardless of genotype, age, or cultivation site, as, on average, root depth represented only 43% of the tree height. The greatest symmetry between vertical growth above and below ground was observed at three years of age; the maximum root depth represented 49%, 29%, 34%, and 34% of the tree height of *E. urophylla* and 61%, 34%, 45%, and 43% of the tree height of *E. grandis* × *E. camaldulensis* at three and six years of age at the wet and dry sites, respectively (Figure 3).

### 2.4. Climate and Its Influence on the Distribution and Depth of Fine Roots

**Figure 4:**
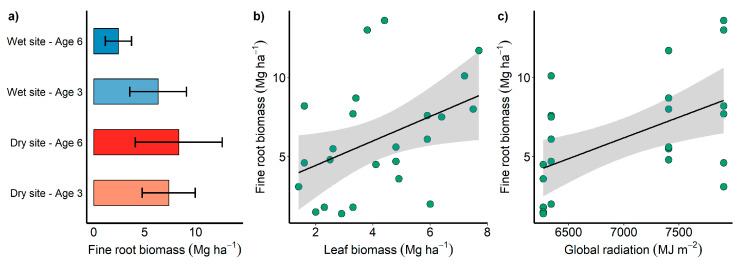
Mean values of the fine root biomass stock (Mg ha^−1^) at three and six years at the dry and wet sites (**a**); relationship between the fine root biomass stock (Mg ha^−1^) and leaf biomass stock (Mg ha^−1^) (**b**); and relationship between the fine root biomass stock (Mg ha^−1^) and global radiation (MJ m^−2^) (**c**).

The GLM indicated that the fine root biomass stock was significantly influenced only by the combination of site and age (Figure 4a), with no influence of genotype. Notably, the highest fine root biomass stock, i.e., 11.42 Mg ha^−1^, occurred at six years of age at the dry site (Figure 4a). There was a significant positive correlation between the fine root biomass stock and leaf biomass stock (Figure 4b) and global radiation (Figure 4c). This indicates that the fine root biomass stock tends to be higher in trees with greater leaf biomass, especially when grown in locations with a higher incidence of global radiation (Figure 4).

**Figure 5:**
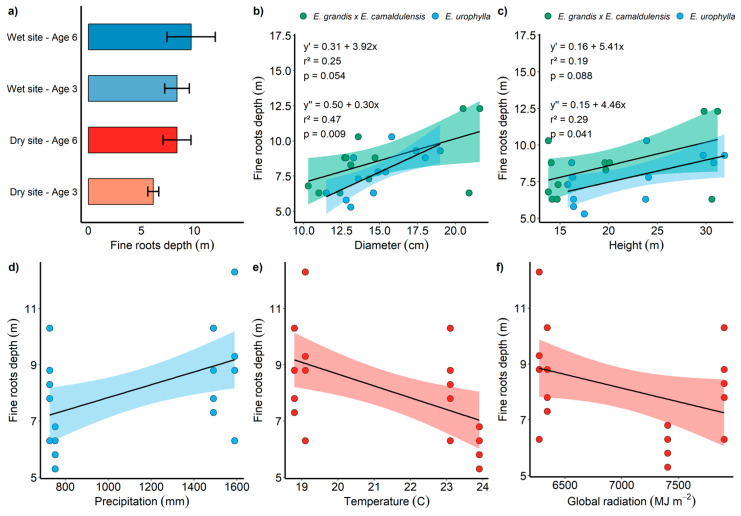
Mean values of the maximum root depth (m) of *E. urophylla* and *E. urophylla* × *camaldulensis* genotypes at the wet and dry sites (**a**); relationship between the maximum root depth and diameter at breast height (1.30 m) for the *E. urophylla* × *camaldulensis* (y′) and *E. urophylla* (y″) genotypes (**b**); relationship between the maximum root depth and total tree height of the *E. urophylla* × *camaldulensis* (y′) and *E. urophylla* (y″) genotypes (**c**); relationship between the maximum root depth and precipitation (**d**); relationship between the maximum root depth and temperature (**e**); and relationship between the maximum root depth and global radiation (**f**).

Similar to the finding for the fine root biomass stock, the GLM demonstrated the influence of site on the maximum root depth (Figure 5a). Furthermore, we found that this variable was positively influenced by precipitation, diameter, height, stem biomass stock, and coarse root biomass stock. On the other hand, variables such as the average temperature, global radiation, and leaf biomass stock were negatively correlated with the maximum root depth (Figure 5).

## 3. Discussion

**Figure 6:**
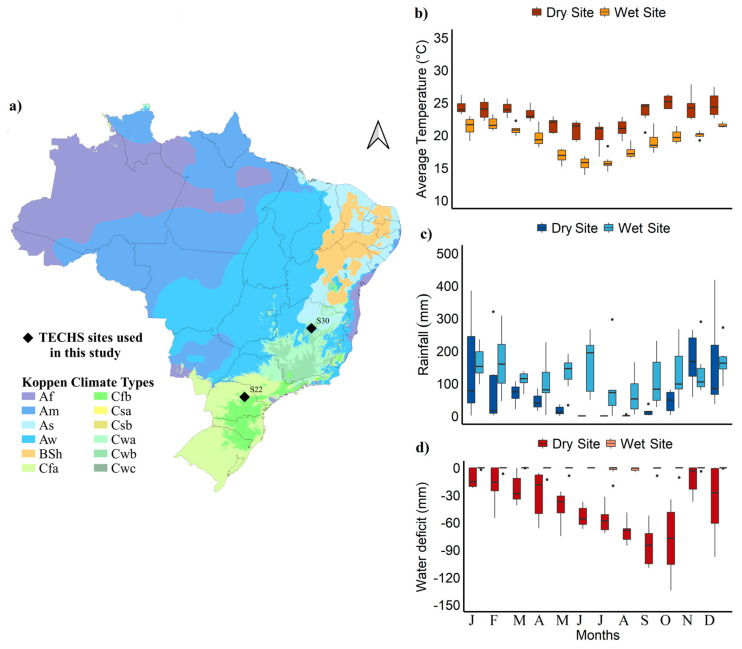
Location of the study sites, adapted from [21] (**a**) and the mean temperature values (°C) (**b**), precipitation (mm) (**c**), and soil water deficit (mm) (**d**) of the sites located in Bocaiuva (S30) and Telêmaco Borba (S22) for the period of January 2012 to December 2017.

Forest growth is commonly described as a function based on the availability of resources such as water, light, and nutrients, the proportion of resources captured by trees, and the efficiency with which trees use resources to fix carbon dioxide [22]. Among the available resources, water is one of the factors that most strongly affects tree growth [23,24], which is consistent with the results found in this study as the highest carbon stocks were measured at the site with greater water availability (Table 1) (Figure 6). Furthermore, *E. urophylla* had higher carbon stocks (90.1 Mg C ha^−1^) than *E. grandis* × *E. camaldulensis* (72.2 Mg C ha^−1^), demonstrating the greater efficiency of this genotype in utilizing the available resources at the site for carbon fixation (Table 1). Similar results were found by [17], who studied the growth behavior of these two genotypes along an edaphoclimatic gradient; at that time, *E. urophylla* exhibited carbon fluxes about 10% higher than those of *E. grandis* × *E. camaldulensis*.

The values of the fine root biomass stock ranged from 0.8 to 5.7 Mg ha^−1^, with a significant influence of site resource availability and age (Table 1) (Figure 5). These values are similar to those found by [9], who studied the fine root biomass stock of four different *Eucalyptus* spp. two years after planting; the fine root biomass stock ranged from 2.2 to 3.7 Mg ha^−1^. Although fine roots represent a small fraction of the total biomass stock, at around 7%, they play a crucial role in carbon and nutrient cycling and accumulation in forest ecosystems [25]. Therefore, understanding their variation among genotypes cultivated at sites with contrasting edaphoclimatic conditions gives us the opportunity to select genotypes that promote denser and deeper root systems, especially in locations with lower resource availability. The expression of plant functional traits can be adjusted to the environment, allowing plants to survive and grow under various climatic conditions [26].

This intraspecific plasticity in response to resource availability was widely observed in the fine root biomass stocks of *E. urophylla* (Figure 1). In soils with reduced water availability (annual rainfall~727 mm), the fine root biomass stock increased from three to six years of age, while the opposite behavior was observed at sites with increased annual rainfall (~1590 mm) (Figure 6). In contrast, no variation was observed in the fine root biomass stock of *E. grandis* × *E. camaldulensis* with respect to age and cultivation site (Figure 1). These results strengthen the relationship between the growth characteristics of the genotypes and those of the species of origin. *E. camaldulensis* is one of the parent species of *E. grandis* × *E. camaldulensis* and is native to regions of Australia with an annual precipitation of 250 to 650 mm, concentrated in four months of the year [27]. In Brazil, *E. camaldulensis* has been cultivated in regions with low soil water availability. Due to its origin in regions with low precipitation levels, *E. camaldulensis* has developed growth strategies, such as the greater allocation of carbon to belowground components, in order to explore a larger volume of soil and reach available water reservoirs to meet the necessary demand for plant growth [17,28].

The fine roots of *Eucalyptus* were primarily concentrated in the shallow soil layers, especially within the first meter of soil (Figure 2). Several factors favor the distribution of superficial roots, such as a lower energy cost for their construction, maintenance, and resource absorption, lower soil resistance to penetration, higher water availability (as most water enters the soil from the surface), and greater oxygen availability [29]. Furthermore, this soil region is characterized by organic and mineral horizons [30] with high nutrient availability, whether from litter decomposition or supplementation through management [31]. Consequently, with increasing soil depth, the density of fine roots decreased exponentially (Figure 4), confirming the theory that the distribution of fine roots along the soil profile is closely related to the availability of resources such as water and nutrients [32]. The greater concentration of fine roots in the superficial soil layers, with a subsequent exponential reduction, demonstrates a standard behavior for different species of the *Eucalyptus* genus regardless of the cultivation site [8,12,33,34].

In addition to the influence of nutrient availability on the density of fine roots in the shallow soil layers, our results demonstrated that the fine root density was also higher for both genotypes at the site with reduced water availability (Figure 2). This reflects another characteristic of these genotypes to increase water absorption during rainy periods as water predominantly enters the soil profile from the surface and subsequently drains to deeper layers. Furthermore, hydrological studies have shown that in semi-arid environments, shallow root systems can be efficient in water absorption [35] as the average depth of the root system is closely related to the average depth of water infiltration into the soil, which mainly depends on the temporal distribution of precipitation at the cultivation site [36]. These results suggest that tree root profiles can be as shallow as possible and as deep as necessary to meet evapotranspiration demands [37].

Furthermore, it is noteworthy that by the age of three, the root system of both genotypes was already fully formed as the maximum average depth reached by the fine roots corresponded to 73% of the maximum average depth achieved at six years (10.0 m) (Figure 3). This can be explained by the high water demand of *Eucalyptus* trees, requiring the extraction of large quantities of water stored in deep soil layers after the previous forest was cut [9]. A rapid detachment of the root front in deep soil layers (6–7 m) in the early years after planting, with a consequent reduction in the root front velocity with age, was also found by [8] when evaluating the distribution of fine roots in *E. grandis* plantations in Brazil.

Although several researchers report greater root depths in dry soils as an alternative to accessing water in deeper soil layers [38], such behavior did not occur in this study. The higher occurrence of fine roots in deep soil layers was positively correlated with precipitation (Figure 5). Additionally, trees with higher growth rates, based on the height, diameter at 1.3 m above ground, stem biomass stock, and coarse root biomass, exhibited greater root depths. This demonstrates that water availability is not the only abiotic factor influencing root depth; factors such as soil texture, the depth of the organic horizon [39], and intrinsic species characteristics also determine the achieved root depth [40]. Another aspect to consider is that existing studies on fine roots are generally conducted in a single environment with good resource availability, comparing genetically productive materials [9,41]. In contrast, this study evaluated different environments with completely contrasting resource quantities (water, light, and nutrients, among others), which directly impacted plant growth.

Overall, the analysis of root depth and density dynamics in *Eucalyptus* plantations over six years, spanning the middle and end of the rotation, revealed notable patterns. The concentration of fine roots in the superficial soil layers during the early years suggests an efficient adaptive strategy to optimize the absorption of nutrients, water, and essential resources. Remarkably, by three years, the root system was already fully formed, representing 73% of the maximum depth reached at six years of age. This phenomenon can be explained by the high water demand of *Eucalyptus* spp., which extract large quantities of water stored in deep soil layers after the previous forest is cut. The rapid initial advance of the root front into deep layers, followed by a deceleration over time, reflects the dynamic adaptation of roots to efficiently search for resources. In line with this study’s conclusion, understanding these patterns in root dynamics in *Eucalyptus* plantations over time is crucial for optimizing management practices, selecting adapted genotypes, and promoting the sustainable use of these forest plantations.

## 4. Materials and Methods

### 4.1. Description of the Study Sites

This study was conducted at two experimental sites, with contrasting climatic conditions, belonging to the Cooperative Research Program for Tolerance of *Eucalyptus* spp. to Water, Thermal, and Biotic Stresses (TECHS) of the Forest Research and Studies Institute (IPEF) [21]. The drier and warmer site (S30) was located near Bocaiuva (17.32° S, 43.77° W), with an average annual temperature of 23.1 °C, 727 mm year^−1^ of rainfall, and a soil water deficit of around 553 mm year^−1^ (Figure 6) from 2012 to 2017. The wet site (S22), located near Telêmaco Borba (22.35° S, 46.97° W), had an average annual temperature of 19.1 °C, precipitation of approximately 1590 mm year^−1^, and a soil water deficit of 8 mm year^−1^.

Climate data (minimum, maximum, and mean air temperatures, precipitation, solar radiation, relative humidity, and wind speed) were recorded from 2012 to 2017 using meteorological stations located in the experimental areas. Missing data for the study period were derived using the nearest meteorological stations of the National Institute of Meteorology (INMET), National Water Agency (ANA), and daily gridded climate data sources such as “CHIRPS” (Climate Hazards Group InfraRed Precipitation with Station) [42], “XAVIER” [43], and NASA POWER (the NASA Langley Research Center POWER Project) [44], as described in [45]. Soil water availability was calculated on a monthly scale using the climatological water balance proposed by Thornthwaite and Mather, modified by [46]. Potential evapotranspiration, that is, the loss of water by soil and plants, was calculated using the Penman–Monteith method parameterized by the FAO [47]. Meanwhile, the soil water storage capacity (SWC) was estimated based on the silt and clay content of the soil at each site [48]. Thus, it was possible to determine the months in which each site exhibited soil surplus (rainfall > evapotranspiration) or water deficit (rainfall < evapotranspiration).

**Figure A1:**
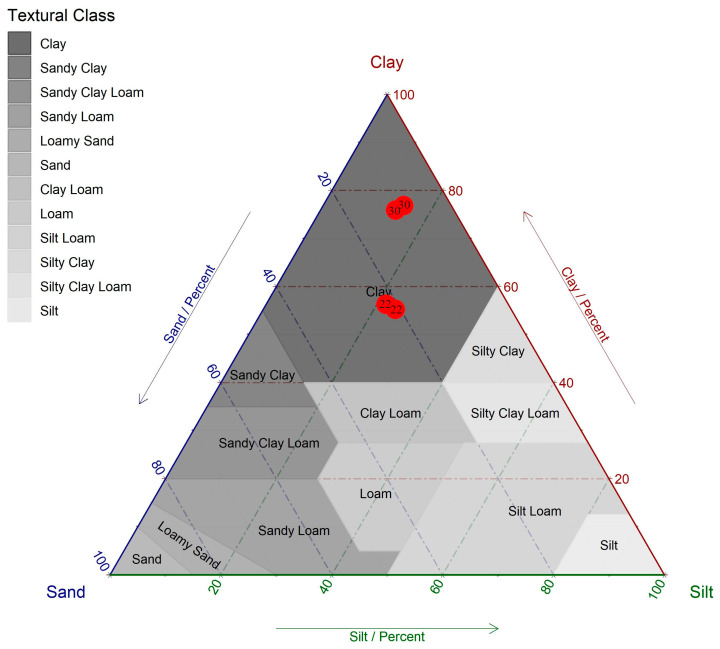
Textural characterization of soil from sites located in Bocaiuva (S30) and Telêmaco Borba (S22). The points represent the soil classification for the depths of 0–20 and 0–40 cm.

The soil texture characterization, i.e., the clay, silt, and sand contents, was conducted following the methodology proposed by [49], specifically for the top soil layers (0–20 cm and 20–40 cm), which are typically used for complementary nutritional recommendations (Table 2). The soil at the study sites is classified as a Latosol [50], with the dry site showing a lower sand content (10%) compared to the wet site (21%) (Figure A1). It is worth noting that both areas were previously used for *Eucalyptus* cultivation.

**Figure A2:**
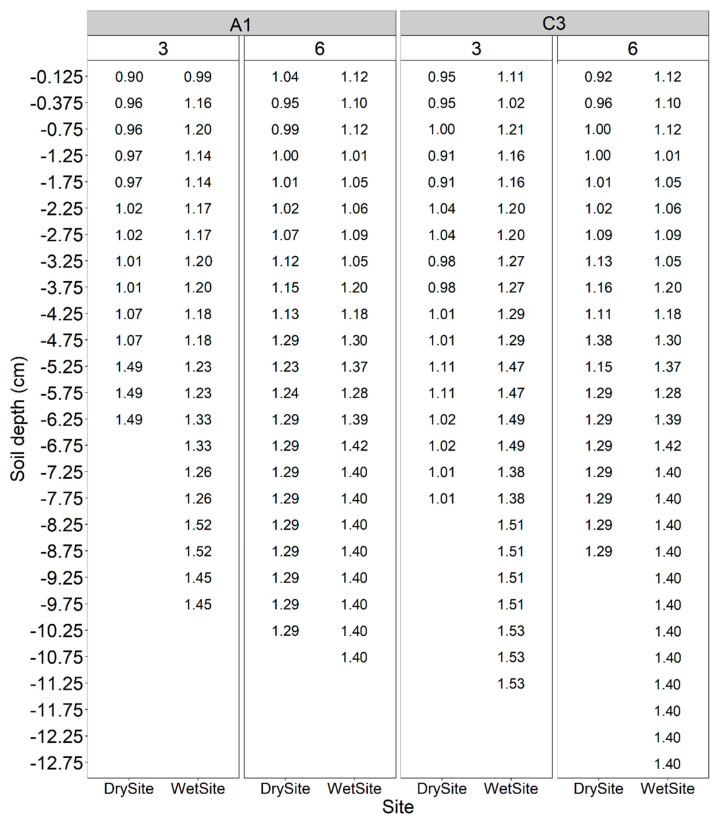
Soil bulk density values (g cm^−3^) at different depths for the sites located in Bocaiuva (S30) and Telêmaco Borba (S22).

The bulk density values were determined for all soil layers in which fine roots were found using samples collected for the quantification of the fine root density. The bulk density of the soil was determined according to the methodology proposed by [51], which aims to measure the average density of a known volume of soil. The highest soil bulk density values were observed at the wet site compared to the dry site across the entire soil profile (Figure A2).

The water holding capacity (WHC) was determined for the top soil (0–40 cm) following the methodology proposed by [52]. The average water retention capacity of the soils ranged from 214 to 225 L m^−2^ at the wet site and the dry site, respectively (Table 2). The wet site had a higher average organic matter content than the dry site; the values were 52 and 47.5 g L^−1^, respectively.

The contents of phosphorus (P), potassium (K), calcium (Ca), and magnesium (Mg) were extracted using ion exchange resin [53]. The levels of organic matter (OM), pH, and cation exchange capacity (CEC) of the soil at the sites were determined following the methodology proposed by [51]. Overall, the highest values for Ca, Mg, and CEC were found in the wet site, whereas for OM, pH, P, and K, the highest values were found in the dry site (Table 2).

The plantations were established in single experimental plots of 0.2 hectares at a spacing of 3 × 3 m in Bocaiúva (dry site) and Telêmaco Borba (wet site) between December 2011 and March 2012. The plots consisted of 8 rows with 15 trees each; the first five trees in each row (border) were used for destructive sampling. The experimental areas were prepared by subsoiling and fertilized with 70 kg N ha^−1^, 45 kg P ha^−1^, 85 kg K ha^−1^, 500 kg Ca ha^−1^, 90 kg Mg ha^−1^, 40 kg S ha^−1^, 3 kg Bo ha^−1^, 1 kg Cu ha^−1^, and 1 kg Zn ha^−1^. Pre-planting fertilizers were applied in straight lines, along with subsoiling, while subsequent applications were applied throughout the canopy expansion, varying from two to four times depending on the region [21].

The plots were planted with *E. urophylla* and *E. grandis* × *E. camaldulensis* clonal seedlings, which are commercially planted by companies in the forestry sector. *E. urophylla* was selected for the Cwa climate (a subtropical climate with dry winters and hot summers) and is one of the most widely planted genetic materials by forestry companies due to its high growth rates across a wide range of locations. On the other hand, the *E. grandis* × *E. camaldulensis* genotype is adapted to the As climate region, and it is generally cultivated in drier areas since its lower growth rates correspond to greater drought resistance [28].

### 4.2. Fine Root Sampling

Fine roots were sampled three (January 2014) and six years (February 2017) after planting at three positions within the plot. The sampling points were located at 0.4 m, 0.9 m, and 1.5 m from three distinct trees, which were previously selected to cover the different diameter classes of the plot. It is worth noting that a minimum distance of 10 m was maintained between sampling positions. In total, 24 points (2 sites × 2 ages × 2 genotypes × 3 positions in the plot) were sampled. Due to the high cost of fine root sampling, we sampled a relatively low number of points within the plot in order to concentrate efforts up to the maximum root depth as most studies conducted in *Eucalyptus* plantations focus on the first few meters of soil.

Soil samples were collected from the 0–0.25-m and 0.25–0.50-m layers and then at every 0.5 m until the maximum depth where fine roots were found using a cylindrical auger with a length of 30 cm and a diameter of 9 cm. To prevent the contamination of soil samples by roots from upper layers, after 2 m depth, we increased the diameter of the hole in the soil and installed a 2-m-long and 20 cm diameter plastic tube. Thus, only soil blocks from the inner part of the auger were used for fine root quantification, and all soil fragments from the upper soil layers were systematically discarded. Soil samples were systematically collected to an additional 2 m depth at each sampling position, and the absence of roots below the layer identified as the maximum root depth was verified. It is worth noting that the absence of any physical or chemical barrier to root growth to a depth of 14 m was verified at all sampling positions. Soil samples were placed in plastic bags, labeled, and stored in freezers at 4 °C until processing, which began approximately 5 days after sample collection [9,41].

Soil samples collected in the field were manually homogenized and weighed at the time of fine root separation. Gravimetric soil water content was determined using 20 g of soil dried at 105 °C until a constant weight was obtained. All the fine roots in the samples were washed with running water using sieves with mesh sizes between 0.5 and 1.19 mm; subsequently, all live roots with lengths > 1 cm were carefully separated by hand. For this study, only live fine roots were quantified, i.e., roots with diameters less than 2 mm, yellowish in color, and flexible; dead roots were discarded. A subsample (10% by weight of each sample) was used to estimate the mass of extremely fine roots (length < 1 cm). Thus, 100% of roots larger than or equal to 1 cm in length and 10% of roots less than 1 cm in length were separated, and we estimated the total amount of roots less than 1 cm in the entire sample [9,41]. Finally, the roots were dried for 24 h at 65 °C and weighed (±0.1 mg). The dry mass of fine roots separated using tweezers (length > 1 cm) and the mass of roots < 1 cm estimated in relation to the subsample were totaled for each soil layer. The fine root mass (g m^−2^) was estimated from the determination of root density (dry root mass in relation to dry soil mass) (g kg^−1^ soil) and soil bulk density (Figure A2).

### 4.3. Sampling of Coarse Roots, Leaves, Branches, and Stems

At three and six years of age, the leaf, branch, and stem biomass stocks were determined destructively using seven trees, which were previously selected to cover the range of diameter at breast height (DBH, i.e., 1.3 m above the ground) within the plot. Out of the seven trees selected for aboveground sampling, four were selected for coarse root sampling, during which the soil from the base of the trunk was excavated to allow the complete removal of the coarse roots (diameter greater than 2 mm). The wet weight of all components was measured in the field, and representative samples (±300 g) were dried at 65 °C until a constant weight was reached in order to determine the dry mass. The amount of carbon stored in the biomass was estimated by multiplying the dry biomass by a factor of 0.5, as proposed by the (IPCC, 2006), which assumes that dry biomass contains approximately 50% carbon. 

### 4.4. Data Analysis

The fine root density, tree height, maximum root depth, and biomass stock of the leaves, wood, and roots at each site were subjected to a generalized linear model (GLM). When significant differences were detected between the combination of categorical variables, i.e., site and age, for each genotype separately, the data were submitted to Tukey’s test at a 5% probability level (*p* ≤ 0.05) to compare the treatment means. The Shapiro–Wilk test was applied to check the normal distribution of residuals. Finally, the influence of climate, soil, and other plant components on fine root density (FRD) and maximum root depth (DFR) was modeled using simple generalized linear models considering the effects of genotype (GE) (*E. urophylla* and *E. grandis* × *E. camaldulensis*), age (AG) (3 and 6 years), mean temperature (Tmed), relative humidity (RH), radiation (Ra), precipitation (PP), soil water availability (WD), soil density (SD), diameter (DI), height (HE), stem biomass (WB), leaf biomass (LB), and coarse root biomass (BCR). Initially, a global model was constructed in the form of Y~SI_AG*GE+ Tmed+RH+Ra+PP+WD+SD+DI+HE+WB+LB+BCR. From the global model, submodels with combinations of uncorrelated variables (r < |0.06|) were obtained using the dredge function of the MuMIn package [54]. Based on the best models, multi-model inference was conducted [55] using the model.avg function of the MuMIn package to calculate the average values and significance of the explanatory variable coefficients. All analyses were performed using R software (version 4.0.0) [56].

## 5. Conclusions

The *E. grandis × E. camaldulensis* genotype exhibited greater root depths regardless of the cultivation site; moreover, the greatest root depths were found at the wet site, which received higher annual rainfall. Greater stocks of fine root biomass were found at the dry site for *E. urophylla*, while for *E. grandis × camaldulensis*, no significant differences were found between different sites and ages.

Our findings provide valuable insights for forest managers and policymakers aiming to optimize *Eucalyptus* plantation management practices for different environmental conditions. By understanding how *Eucalyptus* genotypes adapt to varying edaphoclimatic conditions, stakeholders can make informed decisions to enhance forest productivity, carbon sequestration, and resilience to climate change.

## Figures and Tables

**Table 1:** Distribution of the biomass and carbon stocks in the leaves, wood (stem + branches + bark), coarse roots, and fine roots (<2 mm) of the *E. urophylla* and *E. grandis* × *E. camaldulensis* genotypes at three and six years of age when grown on dry and wet sites. Different lowercase letters indicate statistical differences for the combined genotype and site variables at 5% probability.

Genotype	Site	Age	Leaves		Wood		Coarse Roots		Fine Roots	
			Mg ha^−1^	Mg C ha^−1^	Mg ha^−1^	Mg C ha^−1^	Mg ha^−1^	Mg C ha^−1^	Mg ha^−1^	Mg C ha^−1^
*E. urophylla*	Dry	3	6.7 ± 1.6 a	3.3 ± 0.8	49.7 ± 8.3 c	24.9 ± 3.7	9.7 ± 1.6 c	4.8 ± 0.7	8.4 a ± 3.1 b	4.2 ± 1.4
		6	3.8 ± 0.5 b	1.9 ± 0.3	108.8 ± 9.6 b	54.4 ± 4.3	27.4 ± 7.3 ab	13.8 ± 3.3	11.4 ± 3.2 a	5.7 ± 1.5
	Wet	3	6.5 ± 0.6 a	3.3 ± 0.3	75.6 ± 7.1 bc	37.8 ± 3.1	13.2 ± 1.7 bc	6.6 ± 0.8	6.5 ± 4.1 ab	3.3 ± 1.9
		6	2.5 ± 0.7 b	1.3 ± 0.3	170.6 ± 22.6 a	85.3 ± 10.0	34.3 ± 8.3 a	17.2 ± 3.8	1.7 ± 0.2 b	0.8 ± 0.1
*E. grandis* × *E. camaldulensis*	Dry	3	2.8 ± 0.5 bc	1.4 ± 0.3	36.6 ± 4.5 c	18.3 ± 2.0	10.2 ± 2.0 b	5.1 ± 0.9	6.3 ± 2.1 a	3.2 ± 0.9
		6	1.5 ± 0.1 c	0.8 ± 0.1	56.2 ± 7.6 b	28.1 ± 3.4	26.8 ± 11.2 ab	13.4 ± 5.1	5.3 ± 2.6 a	2.6 ± 1.2
	Wet	3	5.5 ± 0.6 a	2.8 ± 0.3	53.9 ± 4.7 b	26.9 ± 2.1	11.4 ± 0.9 b	5.7 ± 0.4	6.1 ± 1.5 a	3.1 ± 0.7
		6	4.0 ± 1.0 ab	2.0 ± 0.5	156.6 ± 4.3 a	78.3 ± 1.9	35.2 ± 7.8 a	17.6 ± 3.5	3.2 ± 1.6 a	1.6 ± 0.7

**Table 2:** Location of the study sites and the physicochemical characterization of the soils (depth of 0 to 40 cm).

Site	Depth	Clay	Silt	Sand	OM	WHC	pH	P	K	Ca	Mg	CEC
	cm	(%)	(%)	(%)	g L^−1^	L m^−2^	CaCl_2_	mg L^−1^ Soil	mmolc L^−3^			
Dry	0–20	76	14	10	63	225	3.8	4.0	1.4	47.0	25.0	223.4
	20–40	77	15	8	32	225	3.9	0.0	1.8	50.0	28.0	194.8
Wet	0–20	56	22	22	58	214	3.9	5.0	0.9	12.0	11.0	165.9
	20–40	55	24	21	46	214	4.0	2.0	6.7	63.0	11.0	201.7

## Data Availability

Datasets are available upon request to the authors.
